# Enhancing the understanding of parental burnout from an empirical and psychometric perspective

**DOI:** 10.1093/eurpub/ckac130.208

**Published:** 2022-10-25

**Authors:** K Prandstetter, H Murphy, S Olderbak, H Foran

**Affiliations:** 1 Department for Psychology, University of Klagenfurt, Klagenfurt, Austria; 2 Department for Diagnostic and Epidemiology, Institute for Therapy Research, Munich, Germany

## Abstract

**Background:**

Parental burnout (PB) is a relatively new syndrome resulting from chronic parenting stress. Besides efforts to enhance scientific and public understanding of PB, little is known about its link to intimate partner violence (IPV), and the psychometrics of German measures for PB remain an under-researched topic. This study aims to address these gaps by 1) testing the interplay of PB, parenting, couple satisfaction, and IPV, 2) examining the psychometrics of the German version of the Parental Burnout Assessment (PBA).

**Methods:**

Data were collected online as part of an international PB study including Austrian parents aged 18 or older with at least one child at home (0-18). Overall, N = 121 mothers from a community sample reported on family functioning, PB, couple quality and gender roles. Structural equation modeling was applied to assess the fit of the theoretical model. Furthermore, data from N = 220 Austrian and German parents on PB and familial variables during the Covid-19 pandemic were collected to analyze the psychometrics of the German PBA. Confirmatory factor analyses were performed to test the validity of the PBA.

**Results:**

SEM indicated a good model fit, χ^2(37) = 35.51, p = .54; CFI = 1.00; RMSEA = .00, (95% CI = .00 -.06). Furthermore, an indirect effect of couple satisfaction on the link between IPV to PB and IPV to parenting was found.

**Conclusions:**

This study provides preliminary evidence for the importance of couples’ relationship satisfaction in understanding links between IPV and PB as well as parenting in German-speaking mothers. On the basis of these findings, future public health efforts may be organized to focus on preventing PB indirectly or directly by targeting couples’ relationship. Additionally, psychometric results of this study can inform researchers and practitioners about the applicability of the German PBA, improving screening of at-risk parents, and offering support for parents at early stages.

**Key messages:**

- Our findings underscore the importance of couples’ relationships for understanding parental burnout in German-speaking parents.
- Valid and reliable assessment of parental burnout can support the screening of at-risk parents at early stages.

